# Supplementary material for: Transcriptomic signature and pro-osteoclastic secreted factors of abnormal bone marrow stromal cells in fibrous dysplasia
Source: bioRxiv. 2024 Feb 28:2024.02.23.581225. Preprint. [Version 1] doi: 10.1101/2024.02.23.581225 (PMC10962707; doi:10.1101/2024.02.23.581225)
Supplement: Supplement 2 [file NIHPP2024.02.23.581225v1-supplement-2.pdf]

bioRxiv preprint doi: https://doi.org/10.1101/2024.02.23.581225; this version posted February 28, 2024. The copyright holder for this preprint (which was not certified by peer review) is the author/funder. This article is a US Government work. It is not subject to copyright under 17 USC 105 and is also made available for use under a CC0 license.

Supplementary Figures

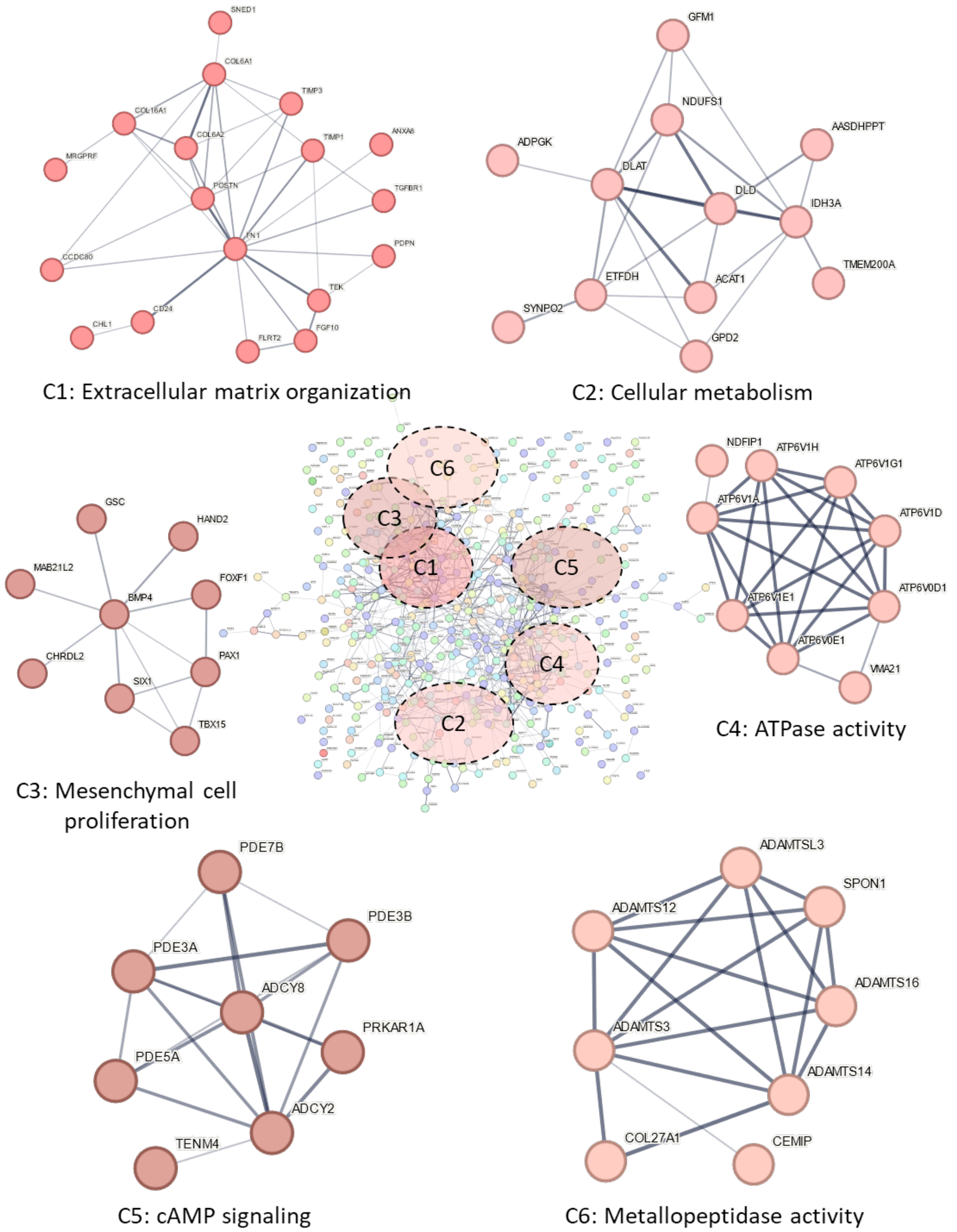

bioRxiv preprint doi: https://doi.org/10.1101/2024.02.23.581225; this version posted February 28, 2024. The copyright holder for this preprint (which was not certified by peer review) is the author/funder. This article is a US Government work. It is not subject to copyright under 17 USC 105 and is also made available for use under a CC0 license.

Figure S1

Pathways affecting several biological processes are enriched by genes found within the FD Signature. Using a statistical threshold of adjusted p<0.01, genes within the signature were loaded into the STRING database to determine potential protein-protein interactions of differentially regulated genes in FD. The most highly enriched processes involved “Extracellular matrix organization” (Cluster 1, C1), “Cellular metabolism” (C2), “Mesenchymal cell proliferation” (C3), “ATPase activity” (C4), “cAMP signaling” (C5), and “Metallopeptidase activity” (C6).

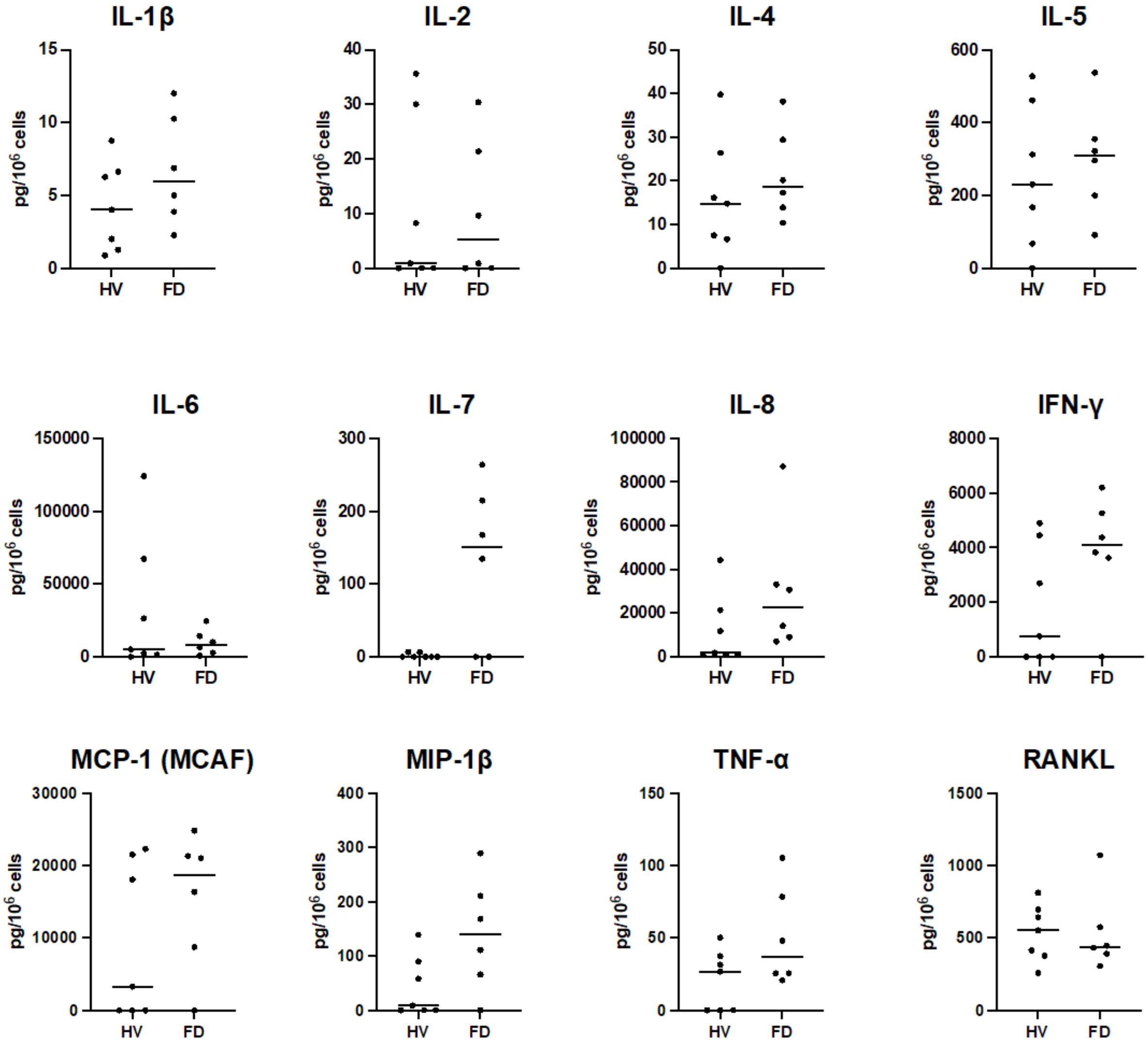

Figure S2

Cultured BMSCs derived from healthy volunteers (HVs) and patients with FD released pro-inflammatory cytokines, but differences were unable to be detected. Additionally, several factors were undetectable (IL-10, IL-12, IL-13, IL-17, G-CSF, and GM-CSF).

bioRxiv preprint doi: https://doi.org/10.1101/2024.02.23.581225; this version posted February 28, 2024. The copyright holder for this preprint (which was not certified by peer review) is the author/funder. This article is a US Government work. It is not subject to copyright under 17 USC 105 and is also made available for use under a CC0 license.

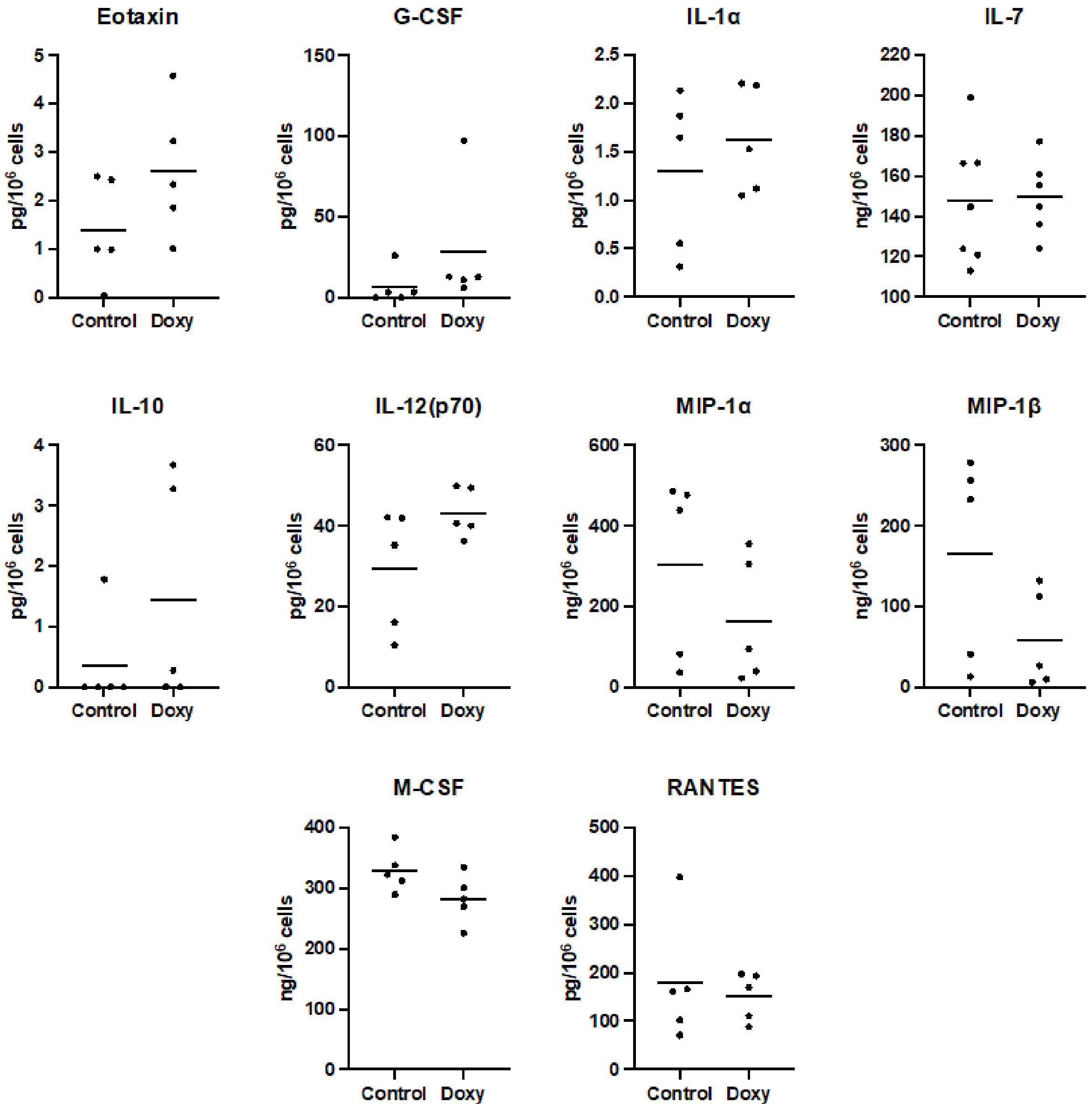

Figure S3

Additional cytokines expressed by cultured murine BMSCs. No differences were detected. IL-4, IL-9 were undetectable.
